# In Vitro Antibacterial and Anti-Inflammatory Properties of Imidazolium Poly(ionic liquids) Microspheres Loaded in GelMA-PEG Hydrogels

**DOI:** 10.3390/gels10040278

**Published:** 2024-04-20

**Authors:** Chao Zhou, Mengdi Sun, Danni Wang, Mingmei Yang, Jia Ling Celestine Loh, Yawen Xu, Ruzhi Zhang

**Affiliations:** 1 School of Medical and Health Engineering, Changzhou University, Changzhou 213164, China; zhouchao@cczu.edu.cn (C.Z.); 2041101204@smail.cczu.edu.cn (M.S.); s22090860022@smail.cczu.edu.cn (D.W.); 2 Department of Dermatology, The Third Affiliated Hospital of Soochow University, Changzhou 213000, China; 20214135026@stu.suda.edu.cn; 3 Department of Dermatology, Affiliated Changzhou Children’s Hospital of Nantong University, Changzhou 213000, China; 4 DUKE-NUS Medical School, National University of Singapore, Singapore 169857, Singapore; e0368666@u.duke.nus.edu

**Keywords:** antibacterial, hydrogel, anti-inflammation, tissue repairing, poly(ionic liquids)

## Abstract

Repairing damaged tissue caused by bacterial infection poses a significant challenge. Traditional antibacterial hydrogels typically incorporate various components such as metal antimicrobials, inorganic antimicrobials, organic antimicrobials, and more. However, drawbacks such as the emergence of multi-drug resistance to antibiotics, the low antibacterial efficacy of natural agents, and the potential cytotoxicity associated with metal antibacterial nanoparticles in hydrogels hindered their broader clinical application. In this study, we successfully developed imidazolium poly(ionic liquids) (PILs) polymer microspheres (APMs) through emulsion polymerization. These APMs exhibited notable antibacterial effectiveness and demonstrated minimal cell toxicity. Subsequently, we integrated the APMs into a gelatin methacryloyl (GelMA)—polyethylene glycol (PEG) hydrogel. This composite hydrogel not only showcased strong antibacterial and anti-inflammatory properties but also facilitated the migration of human skin fibroblasts (HSF) and human umbilical vein endothelial cells (HUVECs) and promoted osteogenic differentiation in vitro.

## 1. Introduction

Tissue repair is the intricate process wherein damaged or deceased local tissues and cells are restored by neighboring healthy cells to regain the integrity of the tissues [1]. However, repairing tissues proves challenging in the presence of bacterial infections, often triggering an inflammatory response. Traditional tissue repair encompasses various categories, including skin tissue repair [2], bone tissue repair [3], vascular repair [4], tracheal repair [5], and more. Anti-bacterial hydrogel scaffolds have emerged as valuable tools in tissue repair due to their ability to efficiently absorb tissue exudate, exhibit high antibacterial activity, maintain low cytotoxicity, and promote cell proliferation and migration [6]. The antibacterial agents designed for tissue repair can be broadly classified into four categories based on their chemical structure and composition: metal antimicrobials [7], inorganic antimicrobials [8], and organic antimicrobials [9], among others.

To overcome the challenges associated with multi-drug resistance in antibiotics, the limited antibacterial efficacy of natural agents, and the potential cytotoxicity of metal antibacterial nanoparticles, researchers have explored the development of various synthesized cationic antimicrobial polymers. Examples include quaternary ammonium, guanidine, *N*-halamine, phosphonium, and sulfonium salt polymers [10]. In a recent study, Feng Yan et al. introduced a groundbreaking antibacterial polymer known as poly(ionic liquids) (PILs), composed of repeating units of ionic liquid (IL) species [11,12,13]. Imidazolium PILs exhibit low toxicity and demonstrate rapid inhibition of both gram-positive and gram-negative bacteria growth [14,15]. The antibacterial mechanism of PILs lies in their positively charged imidazolium group, which disrupts bacterial cell walls by inserting its hydrophobic polymer chain, ultimately fracturing the cell membrane [16,17,18]. Additionally, previous research has indicated that the imidazolium salt group, found in both the small molecular ionic liquids [19,20] and in PILs [14], possesses anti-inflammatory properties. Expanding on this knowledge, our research team has successfully developed various halogen-free imidazolium PIL polymers as highly effective antibacterial agents. These innovative polymers have been incorporated into hydrogels, demonstrating promising applications for infected wound healing [21,22].

When comparing traditional linear antibacterial polymers, antibacterial polymer microspheres (APMs) present numerous advantages, including enhanced drug stability, a larger surface area, higher charge density, structure stability, and low cytotoxicity for cells, among other benefits [23,24,25]. Notably, hydrogel dressings loaded with APMs demonstrate excellent sustained drug release, superior mechanical performance, and potent antibacterial activity [26]. In a study conducted by Xiaohong Hu et al., tetracycline hydrochloride (TH) was encapsulated within gelatin microspheres (GMs) using an emulsion cross-linking method. These GMs were then integrated into an oxidized alginate (OAlg)-carboxymethyl chitosan (CMCS) hydrogel to create a composite hydrogel dressing. The concentrations of GMs in composite hydrogel reached 30 mg/mL, showcasing high compressive strength and effectively inhibiting bacterial growth [27].

**Figure 1:**
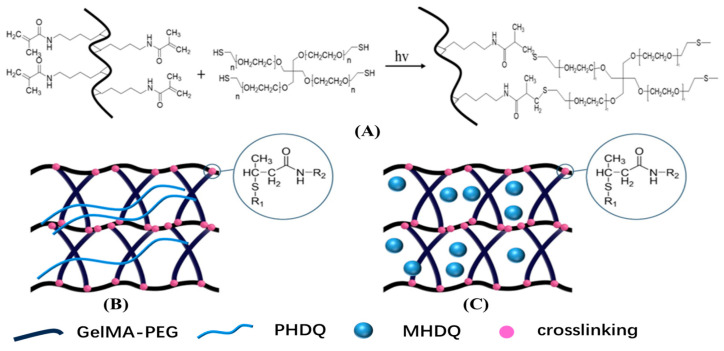
Scheme of (**A**) protocol of GelMA-PEG hydrogel, (**B**) PHDQ-gel and (**C**) MHDQ-gel.

In this investigation, we aimed to enhance the antibacterial activity and anti-inflammatory properties of PILs when incorporated into hydrogels for tissue repair. Firstly, we synthesized APMs through emulsion polymerization, utilizing poly(methacrylamide dopamine) (PDMA), poly(N-(2-hydroxyethyl) acrylamide) (PHEAA), and poly(vinyl imidazolium Bromoethane) (PVIBr). These APMs demonstrated both antibacterial efficacy and low toxicity. Subsequently, composite hydrogels comprising gelatin methacryloyl (GelMA) and polyethylene glycol (PEG) with embedded APMs were fabricated through a thiol-ene click reaction (Figure 1). The resulting composite hydrogels exhibited significant antibacterial and anti-inflammatory properties, along with the ability to promote migration of HSF and HUVEC in vitro, as well as mesenchymal stem cells (MSCs). These findings suggest the potential application of composite hydrogels in tissue repair.

## 2. Results and Discussion

### 2.1. Preparation and Characterization of Linear Antibacterial Copolymers and Microspheres

The linear antibacterial copolymer, denoted as PHDQ, was synthesized through copolymerization of dopamine methacrylamide (DMA), *N*-(2-Hydroxyethyl)acrylamide (HEAA) and 1-Vinylimidazolium bromide (VIBr) monomers. For comparative purposes, a copolymer containing DMA and HEAA was labeled as PHDA, while a copolymer containing DMA, HEAA, and vinyl imidazolium was designated as PHDV (Figure S1a). The molecular weight of these linear copolymers ranged from 3.7 × 10^4^ to 3.8 × 10^4^ g/mol (Table 1).

Moreover, we conducted the synthesis of an antibacterial microsphere (MHDQ) incorporating DMA, HEAA, VIBr, and N,N’-Methylenebisacrylamide (MEAA) through emulsion polymerization. Additionally, microspheres MHDA and MHDV were prepared for comparative analysis (Figure S1b). The FT-IR analysis of MHDQ, in comparison with MHDA and MHDV, is presented in Figure S2. The peaks observed around 1640 cm^−1^, 1560 cm^−1^, and 1375 cm^−1^ corresponded to the stretching vibration of the imidazolium cations, with the peak at 1465 cm^−1^ indicating the vibration of the quaternized cations [28]. The particle sizes are detailed in Table 1. Specifically, the diameter of MHDA was measured at 28 μm, smaller than MHDV (49 μm) and MHDQ (70 μm). The larger particle size of MHDV was attributed to increasing molecular weight after adding 1-vinylimidazolium. Moreover, the particle size of MHDQ still increased after MHDV was quaternized with bromoethane. Furthermore, the zeta potential values in Table 1 indicated that the microspheres exhibited higher values compared to their corresponding linear polymers due to the larger specific surface area of microspheres. Notably, the zeta potential of MHDQ reached 20.9 mV, which is compared to MHDA (5.2 mV) and MHDV (8.92 mV). The reason for the high zeta potential of MHDQ was the larger particle size and more positive charge groups of imidazolium salt. The surface morphology of the microspheres was also examined through FESEM (Figure S3); the MHDQ exhibited larger particle size and agglomeration compared with MHDV and MHDA, and the results were consistent with Table 1.

Table 1 outlines the antimicrobial activity of polymers and microspheres, with the linear polymer PHDQ and microsphere MHDQ demonstrating the most potent antimicrobial effects. Notably, MHDQ exhibited exceptional antibacterial activity, achieving 95.0% inhibition for *E. coli* and 99.8% for *S. aureus*. This remarkable efficacy is attributed to the high positive charge of imidazolium groups, enabling them to bind to the negative charge of bacterial cells, disrupting the cell membrane through electrostatic interactions. Subsequently, the hydrophobic chain on the imidazolium groups further disrupts the bacterial cell membrane, leading to bacterial death [29]. Additionally, the cell viability of both the linear polymers and microspheres, as assessed by CCK-8, exceeded 85% (Table 1).

### 2.2. Structure and Physical Property of Antimicrobial Hydrogels

Hydrogels were formulated through a thiol-ene click reaction, incorporating methacryloyl gelatin (GelMA), four-armed thiol polyethylene glycol (PEG-(SH)_4_) and antibacterial linear polymer/microspheres [30,31]. The resulting antibacterial hydrogel were denoted as PHDQ-gel and MHDQ-gel. Similarly, composite hydrogels, including PHDV-gel, PHDA-gel, MHDA-gel, MHDV-gel, and MHDQ-gel were synthesized using a similar method.

**Figure 2:**
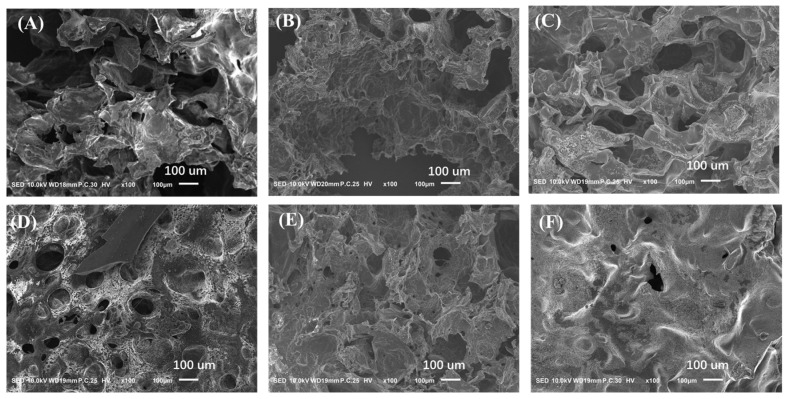
Surface topography of (**A**) PHDA-gel; (**B**) PHDV-gel; (**C**) PHDQ-gel; (**D**) MHDA-gel; (**E**) MHDV-gel; (**F**) MHDQ-gel.

The GelMA-PEG hydrogel, comprising GelMA and PEG-(SH)_4_, served as the control. The FE-SEM images illustrated a porous structure for all hydrogels, as depicted in Figure 2. Notably, the pore sizes of MHDA-gel, MHDV-gel, and MHDQ-gel were observed to be smaller than those of PHDA-gel, PHDV-gel, and PHDQ-gel. The rheological characteristics of the antimicrobial hydrogel were assessed through frequency rheology (Figure S4). The storage modulus (G’) of all hydrogels surpassed their loss modulus (G″), indicating an elastic nature. Particularly, the hydrogels containing antibacterial microspheres exhibited a higher G’ than their G″ at low frequencies.

### 2.3. Antibacterial Activity and Biocompatible of Hydrogels

**Figure 3:**
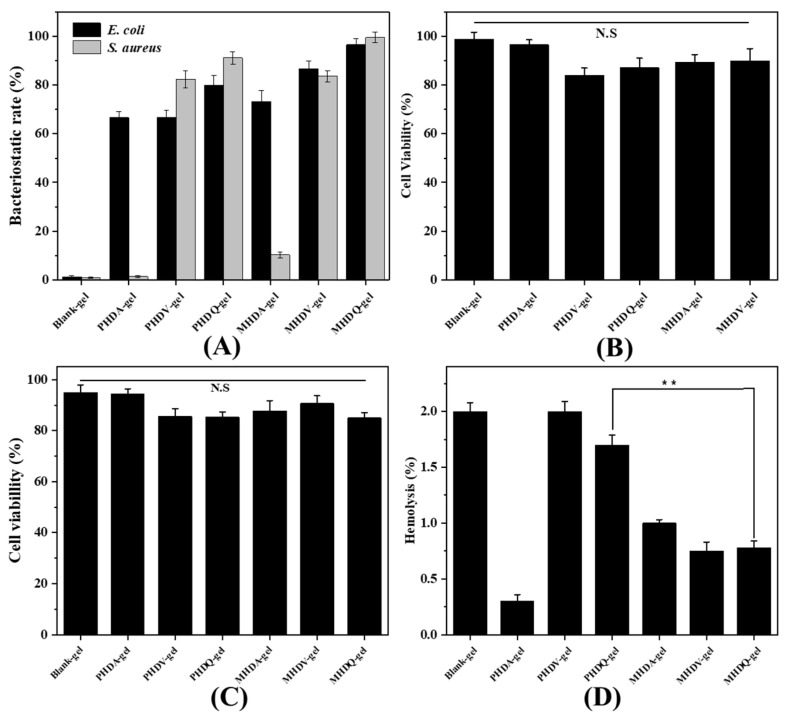
(**A**) Bacteriostatic rate of hydrogels; Cell viability of hydrogels for (**B**) HSF cells and (**C**) U-937 cells; (**D**) hemolysis of hydrogels (** *p* < 0.01).

The efficacy of antimicrobial hydrogels in inhibiting or reducing the growth of *E. coli* and *S. aureus* on their surfaces was assessed (Figure 3A). Over a 24-h period, MHDQ-gel demonstrated notable bacterial inhibition rates, reaching 96.67% against *E. coli* and 99.63% against *S. aureus*, surpassing the performance of the control. In contrast, PHDQ-gel exhibited bacterial inhibition rates of 80.00% against *E. coli* and 91.25% against *S. aureus* within the same timeframe. The remaining hydrogels did not exhibit substantial bacterial inhibition rates against *E. coli* and *S. aureus*, with the superior antibacterial efficacy of MHDQ being a key contributing factor to these results.

The assessment of cytotoxicity on human dermal fibroblasts (HSF) and lymphoma cells (U-937) for the antibacterial hydrogel was carried out using the CCK-8 method, as depicted in Figure 3B,C. The cell viability of HSF and U-937 treated with hydrogel extraction exceeded 85%. Notably, the cell viability of the control reached approximately 99% for HSF and 97% for U-937, underlining the outstanding biocompatibility of gelatin and PEG. While the cell viability of hydrogels containing PHDQ and MHDQ showed a slight reduction compared to the control, it remained above 85%. This indicates the low toxicity of the antibacterial hydrogels to cells, showcasing their potential for applications in tissue repair. To further explore the biocompatibility, the hemolytic properties of the antibacterial hydrogels were tested (Figure 2D), revealing hemolysis levels below 2%, meeting the biomaterial standards outlined in the American Society for Testing and Materials (ASTM) F756-00 standard [21,22].

### 2.4. Anti-Inflammatory Activity of Hydrogels

**Figure 4:**
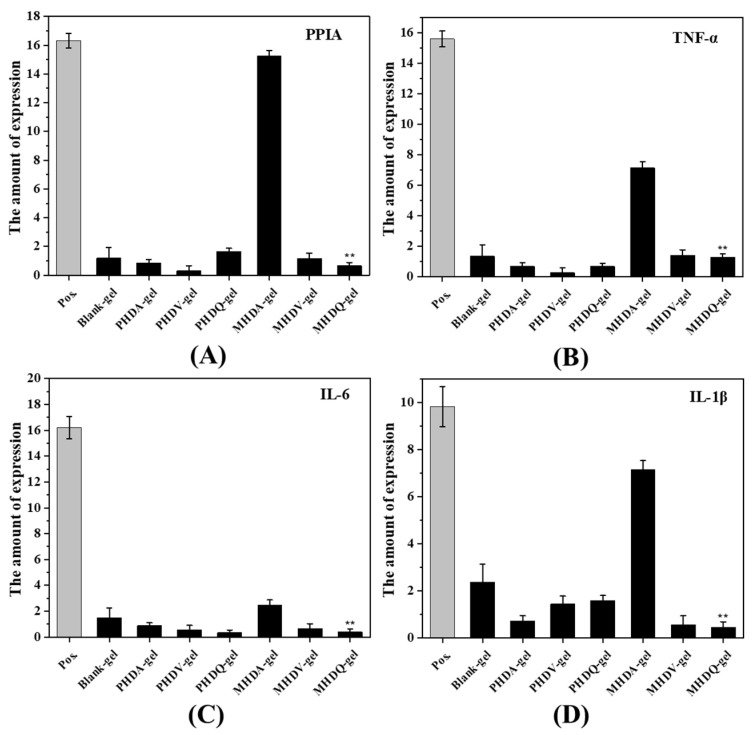
Gene expression of (**A**) IL-1β, (**B**) IL-6, (**C**) PPIA, and (**D**) TNF-α after hydrogel treatment. (** *p* < 0.01).

**Figure 5:**
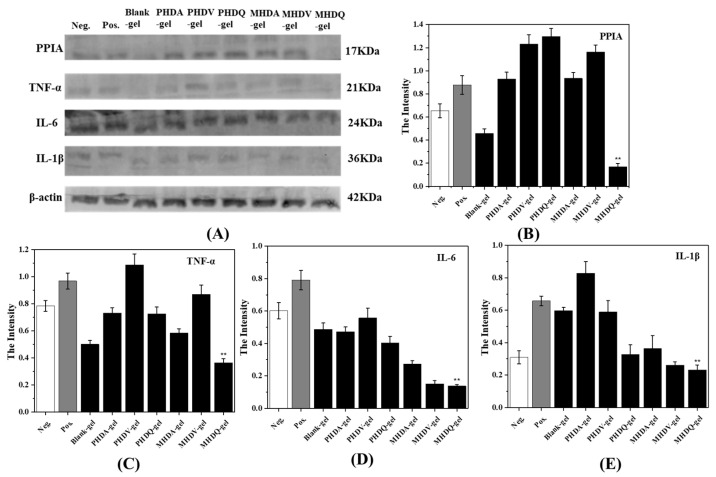
Protein expression test of inflammatory factors by hydrogels with western-blot: (**A**) protein level expression; (**B**–**E**) The intensity for IL-1β, IL-6, PPIA, TNF-α. (** *p* < 0.01).

To ascertain the anti-inflammatory properties of the hydrogels, gene expression analysis (QPCR) and protein expression analysis (Western blot, WB) were performed on U-937 cells for PPIA, TNF-α, IL-6, and IL-1β, respectively. The experimental results, presented in Figure 4 and Figure 5, revealed a downregulation in the expression levels of all four inflammatory factors compared to the positive control group, primarily attributed to the inherent anti-inflammatory effect of gelatin [32]. Notably, MHDQ-gel exhibited the most pronounced downregulation, owing to the anti-inflammatory properties of the imidazolium salt poly(ionic liquid) present in MHDQ-gel. Leveraging the anti-inflammatory characteristics of gelatin, MHDQ-gel amplifies the anti-inflammatory response, leading to a further reduction in the expression levels of inflammatory factors. The notable advantages of this antimicrobial hydrogel in wound dressing applications are particularly evident.

### 2.5. In Vitro Skin Tissue Repairing by Hydrogel Treatment

**Figure 6:**
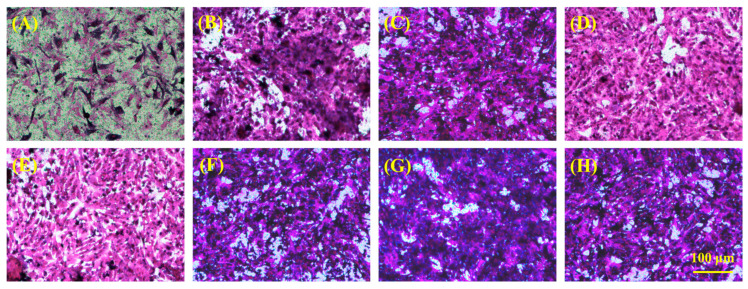
Imaging of cell migration of HSF: (**A**) positive control; (**B**) Blank-gel; (**C**) PHDA-gel; (**D**) PHDV-gel; (**E**) PHDQ-gel; (**F**) MHDA-gel; (**G**) MHDV-gel; (**H**) MHDQ-gel.

**Figure 7:**
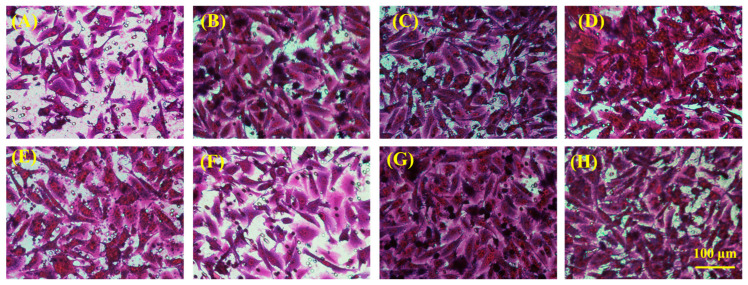
Imaging of cell migration of HUVEC: (**A**) positive control; (**B**) Blank-gel; (**C**) PHDA-gel; (**D**) PHDV-gel; (**E**) PHDQ-gel; (**F**) MHDA-gel; (**G**) MHDV-gel; (**H**) MHDQ-gel.

For the prospective application of hydrogel in skin tissue repair, we employed Transwell technology to assess cell migration, specifically with HSF and HUVEC cells, to observe the effects of hydrogel treatment on cell migration. The experimental outcomes, illustrated in Figure 6 and Figure 7, demonstrated a positive modulatory impact of all hydrogels on the migration of both HSF and HUVE cells, attributable to the inclusion of gelatin in the hydrogels. Notably, hydrogels (PHDQ-gel and MHDQ-gel) incorporating imidazolium salt PILs (PHDQ and MHDQ) exhibited a further enhancement in migration for both HSF and HUVEC cells when compared to positive control groups and other hydrogels. These findings suggest that the antimicrobial hydrogel we developed has the potential to promote skin tissue repair.

### 2.6. In Vitro Bone Tissue Repairing by Hydrogel Treatment

**Figure 8:**
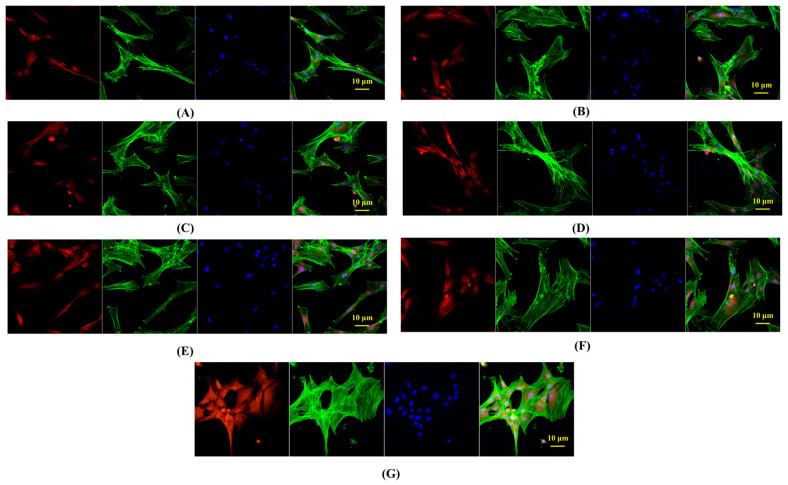
Fluorescence imaging of MSCs by hydrogels treatment: (**A**) Blank-gel; (**B**) PHDA-gel; (**C**) PHDV-gel; (**D**) PHDQ-gel; (**E**) MHDA-gel; (**F**) MHDV-gel; (**G**) MHDQ-gel.

In the context of potential application in bone tissue repair, we conducted observations on the morphological structure and quantity of rat mesenchymal stem cells (MSCs) treated with hydrogels. As depicted in Figure 8A, the number of MSC cells treated with the control was the lowest, exhibiting a slender cell morphology with less evident osteogenic differentiation. Notably, the MSC cell count significantly increased after treatment with PHDQ-gel and MHDQ-gel, as depicted in Figure 8D,G, respectively. Particularly, the MSC cells treated with MHDQ-gel demonstrated the highest cell count, and the presence of a well-defined osteoblast structure indicated a notable phenomenon of osteogenic differentiation.

## 3. Conclusions

In this work, we first synthesized PIL linear polymer PHDQ by radical polymerization and PIL microsphere MHDQ by emulsion polymerization. These materials exhibited robust antibacterial activity while maintaining low cell toxicity. Subsequently, the GelMA- PEG hydrogel was constructed using thiol-ene click reaction, and PHDQ and MHDQ were incorporated into GelMA- PEG hydrogels, respectively. The resulting MHDQ-gel hydrogels not only demonstrated antibacterial and anti-inflammatory properties but also facilitated accelerated migration of HSF and HUVEC, promoting osteogenic differentiation. These findings suggest the potential applications of MHDQ-gel hydrogels in both skin and bone repair.

## 4. Materials and Methods

### 4.1. Materials

N-(2-Hydroxyethyl)acrylamide (HEAA, 99%); N,N’-Methylenebisacrylamide (MEAA, 99%); 1-Vinylimidazole (VI, 99%); 1-Vinylimidazolium bromide (VIBr, 99%); 2,2′-Azobis(2-methylpropionitrile) (AIBN, 99%); 2,2′-Azobis(2-methyl-N-(2-hydroxyethyl)propionamide) (VA-086, 99%); Dopamine hydrochloride (99%); Gelatin (CP); Bromoethane (99%); 3-Mercaptopropionic acid (99%); Triton-X 100 (99%); and Polyoxymethylene (99%) were procured from Aladdin. Four-armed polyethylene glycol (4-PEG, 5K) was obtained from SINOPEG Co. *E. coli* (DH5α) and *S. aureus* (ATCC 25923) were sourced from Beyotime. Human Skin Fibroblast (HSF), Human Lymphoma Cell (U-937), Human Umbilical Vein Endothelial Cells (HUVEC), PRMI-160 Culture Medium, Special DMEM high glucose medium, DMEM Culture Medium, L13152 LIVE/DEAD^®^ Bac LightTM Bacterial Viability Kit, Cell Counting Kit-8 (CCK8), Matrigel Matrix Gel, Lipopolysaccharide (LPS) were purchased from Thermo Fisher Scientific. Rhodamine B and 4,6-Diamidino-2-phenylindole (DAPI) were procured from Cell Signaling Technology.

### 4.2. Synthesis of Antibacterial Copolymers

Dopamine methacrylamide (DMA) was synthesized following a previously reported procedure [33]. Subsequently, the PDMA-PHEAA-PVIBr linear copolymer was synthesized as outlined: HEAA (464 μL, 4.48 mmol), DMA (0.0986 g, 0.448 mmol), and VIBr (0.0909 g, 0.448 mmol) were combined in a 10 mL Slank reaction tube. *N*, *N*-dimethylformamide (3 mL) was added to dissolve the solids. AIBN (0.0015 g, 9.135 × 10^−3^ mmol) was introduced into the reaction tube, and nitrogen gas was bubbled for 10 min. The reaction proceeded at 70 °C in a closed environment for 9 h. Upon completion, the reaction mixture was gradually added to ether under ice bath conditions, leading to the precipitation of solids. After filtration, the residue was vacuum-dried to yield the product, denoted as PHDQ. The copolymers comprising PDMA-PHEAA (PHDA) and PDMA-PHEAA-PVI (PHDV) were synthesized using a method similar to that of the control (refer to Figure S1).

(^1^H NMR (DMSO), PHDA: δ = 1.20 ppm (O=C–CH_3_); δ = 1.63 ppm (–CH_2_–CH_3_); δ = 2.63 ppm (–CH–CH_2_); δ = 3.49 ppm (–NH–CH_2_); δ = 6.50–6.80 ppm (–CH–CH=C–OH))

(^1^H NMR (DMSO), PHDQ: δ = 1.05 ppm (O=C–CH_3_); δ = 1.41 ppm (–CH_2_–CH_3_); δ = 2.45 ppm (–CH–CH_2_); δ = 3.40 ppm (–NH–CH_2_); δ = 6.30–6.64 ppm (–CH-CH=C–OH))

### 4.3. Synthesis of Antibacterial Polymer Microspheres

In a round-bottom flask, under a nitrogen atmosphere at room temperature, N-hexane (70 mL), Span 80 (500 μL), and Tween 80 (100 μL) were combined to create an emulsion, with stirring for 30 min. DMA (0.0986 g, 0.448 mmol), MEAA (0.0414 g, 0.2688 mmol), VA-086 (0.0517 g, 0.1792 mmol), HEAA (464 μL, 4.48 mmol), and VIBr (0.0909 g, 0.448 mmol) were accurately weighed. A mixture of 2.5 mL water and 1.98 mL dimethyl sulfoxide (DMSO) was introduced, and after dissolving the solids, nitrogen purging was performed for 10 min. The mixture was then slowly added to the emulsion, and the reaction proceeded for 4 h under UV light at 365 nm with continuous stirring. Upon completion, the reaction mixture was poured into acetone (300 mL) to induce solid precipitation. The resulting precipitate was filtered, added to isopropanol (30 mL), centrifuged to remove the supernatant, and the process was repeated with acetone (30 mL) and PBS buffer solution. After centrifugation, the supernatant was discarded, and the product was subjected to freeze-drying, yielding a white solid named MHDQ. Polymer microspheres MHDA and MHDV were synthesized using a method similar to that of a control (refer to Figure S1).

### 4.4. Synthesis of Antibacterial Hydrogels

Methacryloyl gelatin (GelMA) and four-armed thiol polyethylene glycol (PEG-(SH)_4_) were synthesized using the methods described in previous publications [30,31]. Subsequently, GelMA (0.05 g), PEG-(SH)_4_ (0.05 g), PHDQ/MHDQ (0.2 g), and VA-086 (0.01 g) were dissolved in 1 mL of PBS, and the solution was purged with nitrogen gas for 2 min. Following this, the mixture was exposed to UV light (365 nm) for 500 s, resulting in the formation of PHDQ-gel/MHDQ-gel. Composite hydrogels, including PHDV-gel, PHDA-gel, MHDA-gel, MHDV-gel, and MHDQ-gel, were synthesized using a similar method. The hydrogel comprising GelMA and PEG-(SH)_4_ served as the control.

### 4.5. Chemical Structure Characterization

The polymer structure and molecular weight were analyzed using Nuclear Magnetic Resonance (NMR) spectroscopy (AVANCE II 500M, Bruker, Karlsruhe, Germany), Fourier Transform Infrared Spectroscopy (FTIR) equipment (NICOLET IS 10, Thermo Fisher SCIENTIFIC, Waltham, MA, USA), and Gel Permeation Chromatography (GPC; PL-GPC 50, Agilent, Santa Clara, CA, USA, with H_2_O as the flow phase and a flow ratio of 1 mL/min).

### 4.6. Zeta Potential and Particle Size Characterization

The copolymers and microspheres were individually dispersed in a PBS buffer solution to create a 0.1 mg/mL solution. Zeta potential testing was carried out on these three sets of samples utilizing a laser particle size analyzer (ZEN 3600, Malvern Instruments Ltd., England, UK). Following this, the microspheres were dispersed in a PBS buffer solution to formulate a 0.1 mg/mL solution. Subsequently, the average particle size of the three microgel particles was determined using a laser particle size analyzer (ZEN 3600, Malvern Instruments Ltd., Malvern, UK).

### 4.7. Rheological Performance and Surface Morphology of Antibacterial Hydrogel

The rheological characteristics of the hydrogels were assessed using the Malvern Kinexus Pro rheometer. Frequency rheological experiments were conducted under a constant oscillatory strain (γ) of 0.5% across a frequency range (F) of 0.1–20 Hz to evaluate the viscosity and elasticity of the antibacterial hydrogel. Additionally, the morphology of the hydrogels was examined using a scanning electron microscope (Zeiss Sigma 500, Oberkochen, Germany).

### 4.8. Determination of In Vitro Biocompatibility of Hydrogels

Cytotoxicity testing of the hydrogels for HSF and U937, which were evaluated by cell counting Kit-8 (CCK-8), and hemolysis testing were adapted from a previously published method [21]. Firstly, the hydrogels were immersed in 5 mL of PBS for 2 h at room temperature. Secondly, 100 μL of the hydrogel extract was mixed with 100 μL of 10^5^ cells/well HSF suspension (cultured in DMEM-H containing 10% FBS and 1% penicillin-streptomycin) or U937 suspension (cultured in RPMI-1640) in a 96-well plate and incubated at 37 °C in 5% CO_2_ atmosphere for 24 h. Then, 10 μL of 10% CCK-8 solution was added to each well; the cells were further incubated for an additional 2 h. Finally, the plate was subjected to OD measurement at 450 nm using a microplate reader (Infinite F50). PBS was used as the blank. Cell viability was calculated by Equation (1):

(1) $$Cell\;viability\;\%=\frac{{OD}_{\mathrm{sample}\;}}{{OD}_{\mathrm{control}\;}}\times 100\%$$

To assess the hemolytic activity of hydrogels, fresh human blood (3 mL) was obtained from an 8-week old mouse (male). Firstly, the blood was then centrifuged at 1200 rpm for 8–10 min using a high-speed centrifuge to separate the red blood cells from the blood plasma. Secondly, the supernatant was carefully aspirated, and the red blood cells were washed with PBS buffer three times, with each wash involving a new centrifugation step. Following the washing process, a 5% *v*/*v* red blood cell solution was prepared using PBS; then the samples were prepared for testing. Thirdly, the extraction of the hydrogels (100 µL, extracted for 24 h) were placed in each well of a 96-well plate, and 100 µL of the red blood cell solution was added to each well. To ensure adequate contact between the sample and the cell solution, the plate was placed on a constant-temperature shaker and shaken at 200 rpm for 1 h. After incubation, the plate was centrifuged again for 8–10 min, and the supernatant was collected. Finally, the absorbance at 540 nm was measured using a microplate reader (Infinite F50). A red blood cell solution dissolved in 0.3% Triton X-100 was used as a positive control and a red blood cell solution without any sample served as a negative control. Each experimental group was measured seven times. The hemolysis percentage was calculated using Equation (2).

(2) $$\mathrm{Hemolysis}\;(\%)=\frac{{\mathrm{OD}}_{\mathrm{sample}}-{\mathrm{OD}}_{\mathrm{negative}}}{{\mathrm{OD}}_{\mathrm{positive}}-{\mathrm{OD}}_{\mathrm{negative}}}\times 100\%$$

where *OD*_sample_ is the absorbance of the samples, *OD*_positive_ is the absorbance of the positive control group, and *OD*_negative_ is the absorbance of the negative control group.

### 4.9. Determination of In Vitro Antibacterial Activity of Copolymer, Microspheres, and Hydrogel

Minimum inhibitory concentrations (MICs) for *E. coli* and *S. aureus* of the copolymers were determined following methods established in previous publications [34]. The antibacterial activity of microspheres was assessed as follows: Initially, *E. coli* and *S. aureus* were separately cultured under shaking conditions at 37 °C with a rotation speed of 100 r/h for 16–18 h to obtain the required original bacterial solution. Subsequently, 0.01 g of each sample was added to 1 mL of the original bacterial solution. A set of bacterial solutions without any added samples was prepared as a blank control. These bacterial solutions were then incubated in a constant temperature incubator at 37 °C for 24 h. Following cultivation, a tenfold gradient dilution method was employed to dilute the bacterial solution five times. Starting from the fifth dilution, 100 μL of the bacterial solution was taken and dropped onto a solid culture medium (plate), which was evenly spread using a triangular spreader. Three parallel samples were prepared. The plated samples were incubated in a bacterial incubator at 37 °C for 24 h. After incubation, the plates were retrieved, and the number of bacterial colonies on each plate was counted. The antibacterial rate of the microgel was calculated using Equation (3).

(3) $$Bacterial\;inhibitiion\;rate\left(\%\right)=\frac{{CFU}_{\left(OD\right)}-{CFU\prime }_{\left(OD\right)}}{{CFU}_{\left(OD\right)}}\times 100\%$$

where, *CFU*_(*OD*)_ is the bacterial count for the control group, *CFU*′_(*OD*)_ is the bacterial count for the sample group. The antibacterial activity of hydrogels was calculated using previous publications [22].

### 4.10. Determination of In Vitro Anti-Inflammatory Activity

#### 4.10.1. Gene Expression by Quantitative PCR

Cultivate U-937 cells in a six-well plate and culture the cells until they reach 70% density. Subsequently, add LPS (1 μL/mL) to each well, incubate at 37 °C for 3 h, then add ATP (10 μL/mL) and incubate for an additional 30 min at 37 °C. After this, add antimicrobial hydrogel extract (100 μL) to each well and culture for 2 h at 37 °C (PBS buffer was added as a positive control group, and cells without LPS, ATP, and antimicrobial hydrogel extract treatment served as the negative control group).

The cultured cells were transferred to a centrifuge tube and centrifuged at 1800 rpm for 5 min. After removing the supernatant, add Trizol reagent for cell lysis (1 mL). Chloroform (200 μL) was added into the centrifuge tube and centrifuged at 14,000 rpm for 8 min. The upper aqueous phase was mixed with isopropanol (500 μL) and centrifuged at 16,000 rpm for 8 min. After removing the supernatant, 70% ethanol was added and centrifuged at 16,000 rpm for 3 min. Finally, DEPC water (10 μL) was added to dissolve RNA, and the RNA concentration was measured using Nanodrop 2000.

For reverse transcription, a mixture of 10× RT mix solution (2 μL), Hiscript III Enzyme mix (2 μL), Oligo dT (1 μL), DEPC water (5 μL), and diluted RNA solution (10 μL) was prepared. The mixed solution was added to the above RNA solution, and reverse transcription into cDNA was performed. For real-time fluorescence PCR, 2× SYBR Green (5 μL), primers (0.25 μL), and cDNA (4.5 μL) were added to each well and centrifuged at 100 rpm for 2 min. Finally, the level of inflammatory factors was characterized by a real-time fluorescence PCR instrument, and the cycle threshold (CT) for the expression level of inflammatory factors was determined using a specific Equation (4).

(4) $$Gene\;Expression=2^{-\left\{\left({C_{T}}_{sample}-{C_{T}}_{reference}\right)-({C_{T}}_{negative}-{C_{T}}_{reference})\right\}}$$

where ${C_{T}}_{sample}$ is the level of inflammatory factors in the antimicrobial hydrogel; ${C_{T}}_{reference}$ is the level of inflammatory factors in the reference; ${C_{T}}_{negative}$ is the level of inflammatory factors in the negative sample.

#### 4.10.2. Protein Expression by Western Blot

U-937 cells were cultured to 80% confluency in each well, followed by the addition of LPS (1 μL/mL) and incubation at 37 °C for 3 h. Subsequently, ATP (10 μL/mL) was added, and the cells were further incubated for an additional 30 min at 37 °C. Next, hydrogel extract (100 μL) was introduced into each well, and the cells were cultured for 2 h at 37 °C. (PBS was utilized as the control group, while cells without LPS, ATP, and hydrogel extract treatment served as the negative control group). The U-937 cells containing samples were then transferred to centrifuge tubes, centrifuged at 1500 rpm for 5 min, and the supernatant was removed. Following this, Western and IP cell lysis solution (100 μL) was added to the centrifuge tube, and the cells were thoroughly lysed with the addition of a protein inhibitor (3 μL). The fully lysed cells were placed on ice for 30 min, followed by centrifugation at 1400 rpm for 3 min to obtain the protein solution. Simultaneously, the SDS-PAGE protein loading buffer was diluted to 1×. Deionized water (60 μL) and protein (20 μL) were combined, and the mixture was water-bathed at 100 °C for 5 min.

The SDS-PAGE gel was prepared, and protein solution (20 μL) along with Marker (3 μL) were added. The gel was electrophoresed at 100 Voltage for 30 min initially and then at 120 Voltage for 45 min. Subsequently, the PVDF membrane was briefly immersed in methanol for 15 s before being placed onto the SDS-PAGE gel. The marker was transferred from the SDS-PAGE gel to the PVDF gel at 300 mA for 90 min. The transferred PVDF membrane was washed in deionized water and immersed in a 5% BSA solution (1 g BSA, 20 mL TBST) for 1 h. The primary antibody solution (1 mL) was applied to the PVDF membrane at 4 °C for 8 h, followed by two washes with TBST. The secondary antibody solution (10 mL) was then added to the PVDF membrane in the dark for 1 h and washed three times with TBST. Finally, the PVDF membrane was scanned to obtain the Western Blot image, and its grayscale intensity (Intensity) was calculated with Equation (5).

(5) $$Intensity=\frac{{Intensity}_{sample}}{{Intensity}_{Marker}}$$

where, ${Intensity}_{sample}\;$ is the intensity of the sample, ${Intensity}_{Marker}\;$is the intensity of the marker.

### 4.11. Determination of In Vitro Tissue Repairing

#### 4.11.1. Cell Migration Assay

To assess the influence of the antimicrobial hydrogel on cell migration, we performed cell migration experiments using human skin fibroblasts (HSF) and human umbilical vein endothelial cells (HUVEC). A suspension of fibroblast or HUVEC cells was introduced into the cell chamber of TSC-003-024, with 250 μL of cell suspension added to each upper chamber. The chambers were then incubated at 37 °C in a 5% CO_2_ atmosphere for 24 h. Following cell attachment, 500 μL of high-glucose culture medium and 100 μL of antimicrobial hydrogel extract were added to the lower chamber, and the cell chambers were continued to be incubated at 37 °C in a 5% CO_2_ atmosphere for an additional 24 h. After incubation, the cells underwent staining. The upper chamber was extracted, and the cells were immersed in a 4% polyformaldehyde solution for 30 min to fix the cells. After removal, the lower membrane cells were stained with crystal violet for 15 min, followed by washing with PBS. Subsequently, a cotton swab was utilized to gently wipe away the cells on the upper side of the membrane that did not pass through, and observation and photography were conducted using an optical microscope.

#### 4.11.2. Immunofluorescence Staining Test

We employed primary rat mesenchymal stem cells (MSCs) isolated from rats’ femurs and tibias for the study. MSCs were co-cultured with a medium containing the extract of the antimicrobial hydrogel on coverslips. Following this, cells were rinsed with PBS and fixed in 4% polyformaldehyde at room temperature for 20 min. Subsequently, permeabilization was carried out with 0.1% Triton X-100 for 10 min, followed by blocking with 5% BSA for 1 h. Primary antibodies targeting AKT (Abcam), OPN (Abcam), and RUNX2 (Abcam) were applied to cells overnight at 4 °C with 3% BSA. Afterward, cells underwent washing with 1×PBS three times and incubation with secondary Alexa Fluor^®^ 594-conjugated antibodies (Abcam) and Alexa Fluor 488-conjugated molecular probe guanidine peptide (Sigma-Aldrich, Missouri, USA) at room temperature for 1 h. DAPI (4′,6-diamidino-2-phenylindole; Sigma-Aldrich) was applied for 5 min, and coverslips were mounted on glass slides. Observation and photography were conducted using a laser scanning confocal microscope (Axio Imager M1; Zeiss, Oberkochen, Germany).

## Figures and Tables

**Table 1:** Characterization of linear polymers and microspheres.

Samples	GPC			Particle Size (nm)	Zeta Potential (mV)	Bacterial Inhibition (%)		Cell Viability (%)
	*M_n_* (g/mol)	*M_w_* (g/mol)	PDI			*E. coli*	*S. aureus*	
PHDA	3.7 × 10^4^	9.1 × 10^4^	2.4	-	0.2	36.1	1.0	88.9
PHDV	3.8 × 10^4^	1.1 × 10^5^	2.9	-	3.2	78.7	73.1	90.0
PHDQ	3.8 × 10^4^	1.1 × 10^5^	2.9	-	10.8	89.3	94.9	85.0
MHDA	-	-	-	28	5.2	54.7	2.9	90.0
MHDV	-	-	-	49	8.92	85.5	82.7	89.9
MHDQ	-	-	-	70	20.9	95.0	99.8	85.0

## Data Availability

The original contributions presented in the study are included in the article/Supplementary Material, further inquiries can be directed to the corresponding authors.

## Supplementary Materials

The following supporting information can be downloaded at: https://www.mdpi.com/article/10.3390/gels10040278/s1, Figure S1: Procedure of synthesis of (A) PHDA, (B) PHDV, (C) PHDQ, (D)MHDA, (E) MHDV and (F) MHDQ; Figure S2: FT-IR of (A) MHDA; (B) MHDV; (C) MHDQ; Figure S3: Surface topography of microspheres: (A) MHDA, (B) MHDV and (C) MHDQ; Figure S4: Frequency scanning of (A) PHDA-gel, (B)PHDV-gel, (C) PHDQ-gel, (D) MHDA-gel, (E) MHDV-gel (F) MHDQ-gel at constant strain γ = 0.5%, F = 0.1–20 Hz.
