# Supplementary material for: In Vitro Antibacterial and Anti-Inflammatory Properties of Imidazolium Poly(ionic liquids) Microspheres Loaded in GelMA-PEG Hydrogels
Source: Gels. 2024 Apr 20;10(4):278. doi: 10.3390/gels10040278 (PMC11048874; doi:10.3390/gels10040278)
Supplement: Supplementary file 1 [file gels-10-00278-s001.zip › gels-2953732-supplementary.pdf]

gels

MDPI

In Vitro Antibacterial and Anti-Inflammatory Properties of Imidazolium Poly(Ionic Liquids) Microspheres Loaded in GelMA-PEG Hydrogels

Chao Zhou [1,†], Mengdi Sun [1,†], Danni Wang [1], Mingmei Yang [2,4], Jia Ling Celestine Loh [3], Yawen Xu [2,*] and Ruzhi Zhang [2,*]

1 School of Medical and Health Engineering, Changzhou University, Changzhou 213164, China; zhouchao@cczu.edu.cn (C.Z.); 2041101204@smail.cczu.edu.cn (M.S.); s22090860022@smail.cczu.edu.cn (D.W.)
2 Department of Dermatology, The Third Affiliated Hospital of Soochow University, Changzhou 213000, China; 20214135026@stu.suda.edu.cn
3 DUKE-NUS Medical School, National University of Singapore, Singapore 169857, Singapore; e0368666@u.duke.nus.edu
4 Department of Dermatology, Affiliated Changzhou Children's Hospital of Nantong University, Changzhou 213000, China
* Correspondence: xyw4937@suda.edu.cn (Y.X.); gy2016@suda.edu.cn (R.Z.)
† These authors contributed equally to this work.

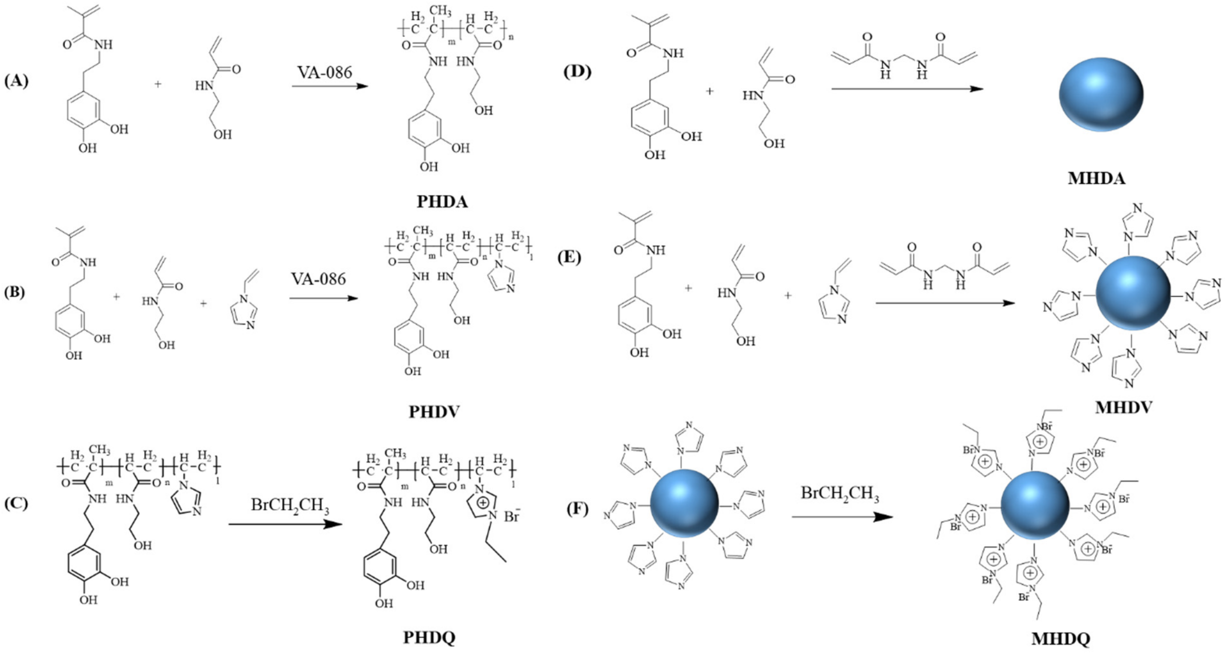

Figure S1. Procedure of synthesis of (**A**) PHDA, (**B**) PHDV, (**C**) PHDQ, (**D**)MHDA, (**E**) MHDV and (**F**) MHDQ.

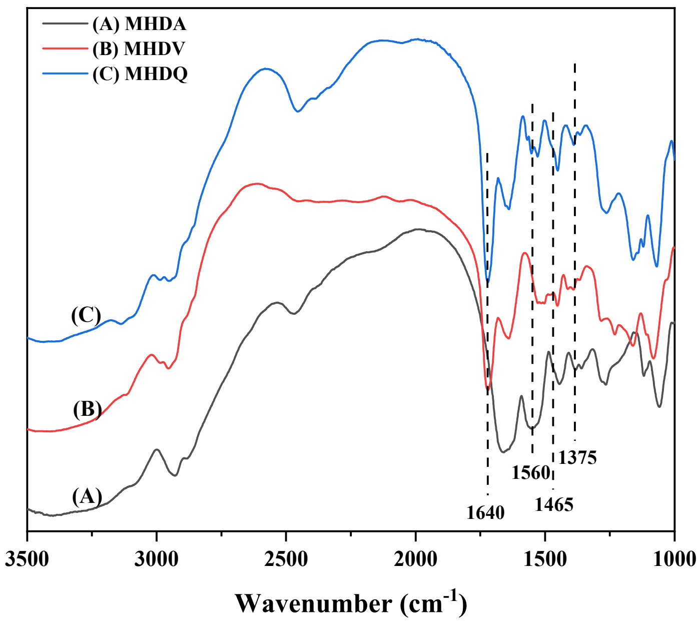

Figure S2. FT-IR of (**A**) MHDA; (**B**) MHDV; (**C**) MHDQ.

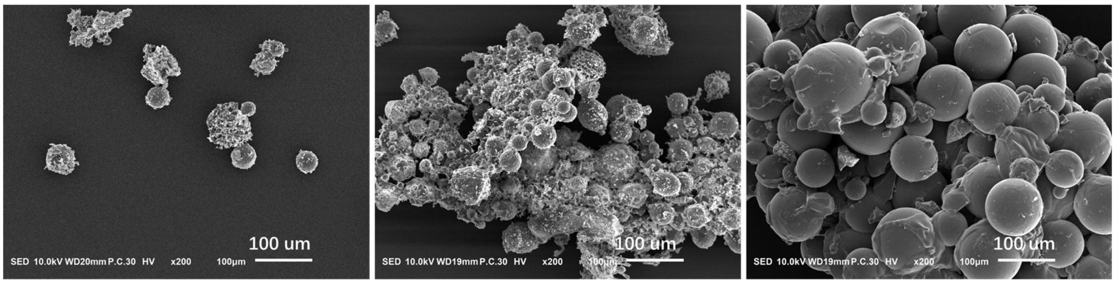

Figure S3. Surface topography of microspheres: (**A**) MHDA, (**B**) MHDV and (**C**) MHDQ.

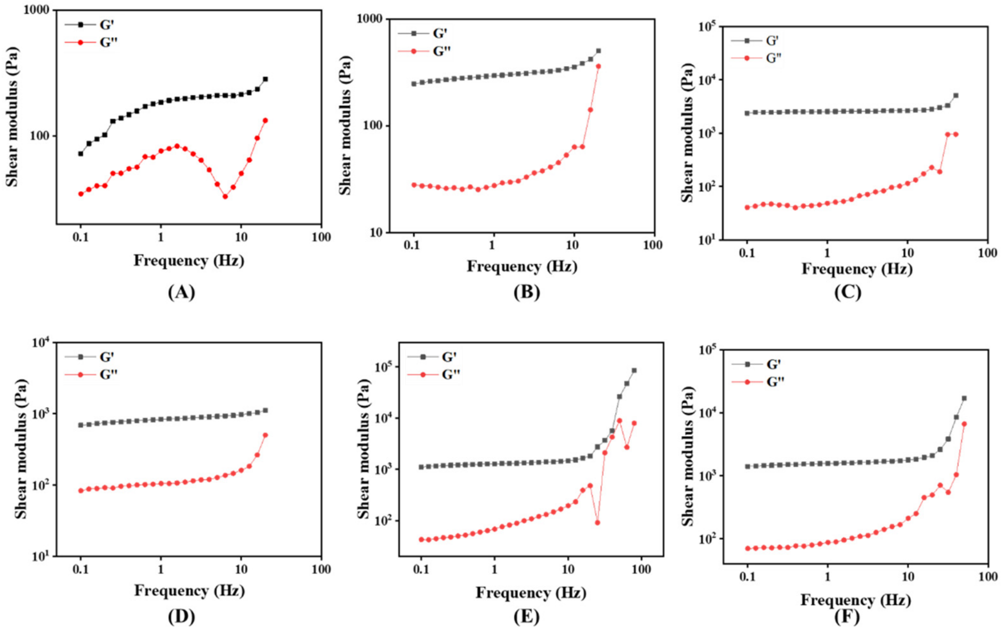

Figure S4. Frequency scanning of (**A**) PHDA-gel, (**B**)PHDV-gel, (**C**) PHDQ-gel, (**D**) MHDA-gel, (**E**) MHDV-gel (**F**) MHDQ-gel at constant strain γ= 0.5%, F= 0.1–20 Hz.
